# Reproductive behaviors among school-based health center clients in New Mexico

**DOI:** 10.3389/frph.2024.1244135

**Published:** 2024-05-07

**Authors:** Mayra Perez, Allyson Kelley

**Affiliations:** ^1^ University of New Mexico Health Sciences Center, Office for Community Health, Community Health Worker Initiatives, University of New Mexico, Albuquerque, NM, United States; ^2^ Allyson Kelley & Associates PLLC, Sisters, OR, United States

**Keywords:** school-based health center, reproductive health, teen, birth control, New Mexico

## Abstract

**Introduction:**

School-based health centers (SBHCs) are an evidence-based model for providing contraceptives to adolescents. SBHCs ability to provide reproductive health services is often limited by school district policies, state laws, and health center policies.

**Methods:**

We used data from the Teens Exploring and Managing Prevention (TEMPO) study to document demographic characteristics and birth control methods among patients at four SBHCs in New Mexico. A total of 264 teens were included in the baseline data collection at SBHCs in New Mexico. A baseline survey was administered via iPad, that specifically focused on questions related to sexual experiences and sexual health topics. Baseline questions included demographic questions, gender identity, sexual orientation, reasons for visits, reproductive practices, and birth control methods. Descriptive statistics were calculated, including means and standard deviations for continuous variables and categorical frequencies.

**Results:**

Our findings present reproductive health behaviors among New Mexican patients attending schools with SBHCs. More than 74% of respondents were Hispanic or Latino. The most common reason teens went to the SBHC was for birth control, and just 62% of teens reported using birth control methods in the past.

**Discussion:**

These behaviors are essential for policymakers to consider as they address policy gaps, the complex landscape of parental permission, reproductive rights, and health as a human right.

## 1 Introduction

School-based health centers (SBHCs) are an evidence-based model for providing contraceptives to adolescents (1). SBHCs play a vital role in providing healthcare services for many underserved populations of children and adolescents in the United States (2). Many SBHC patients lack insurance and may not have regular access to a primary care doctor or health education resources (1). Previous reports indicate that 70% of SBHC patients were racial or ethnic minorities and frequently residents with lower socioeconomic status (3). Offered at elementary, middle, and high schools throughout the country, SBHCs provide a variety of healthcare services on and off school property including sexual health education and reproductive health services (4). While SBHCs are lauded as an effective population-based strategy to support healthy sexual behaviors, their ability to provide reproductive health services is limited by school district policies, state laws, and health center policies (5, 6). A key issue in the provision of services at SBHCs is minors' consent for health care services and the HIPPA Privacy Rule [45 CFR 164.502(g)], which states a parent or guardian has access to their child's medical records. While New Mexico is included as one of the 23 states and the District of Columbia that allow all minors to consent to contraceptive services, the majority of states in the US do not (7).

### 1.1 About New Mexico

New Mexico laws allow healthcare providers (and SBHCs) to provide examinations and services for sexually transmitted diseases, pregnancy, contraception, emergency conditions, and mental health/substance use (8). While one would think that these laws would ease access to reproductive health services and improve reproductive health outcomes in New Mexico, the data tell a different story. Teen birth rates have decreased in the US, and other key health indicators have improved; however, New Mexico lags behind. Key health indicators related to reproductive health in New Mexico underscore the need for reproductive health services. Based on 2020 data, the fertility rate is 54.6 per 1,000 women (15–44 years of age), the teen birth rate is 21.9 per 1,000 births (females 15–19 years of age), and the infant mortality rate is 5.1 per 1,000 (deaths per 1,000 live births) infant deaths (9). Teen birth rates are one indicator of reproductive health services, and New Mexico has the 10th highest teen birth rate in the US (Mississippi is the highest with 27.9 per 1,000, and Massachusetts is the lowest with 6.1 per 1,000) (9).

Place, race, and poverty contribute to high teen birth rates. New Mexico is home to more than 2 million people, and 50% are Hispanic or Latino (10). Poverty is common; 18.4% of residents living below the federal poverty level and a median household income of $54,020 (10) do not have health insurance. SBHCs address poverty and the lack of insurance in New Mexico school-aged residents. As of 2022, there were 78 SBHCs in New Mexico and 52 of these SBHCs are contracted with the Department of Health to bill Medicaid (11). Yet, there have been inadequate studies that examined the reproductive health practices of patients enrolled in SBHCs in New Mexico. Documenting the characteristics and reproductive health behaviors of SBHC patients in New Mexico is the first step in designing effective interventions that promote healthy reproductive practices in youth.

## 2 Materials and methods

We used data from the Teens Exploring and Managing Prevention (TEMPO) study to document demographic characteristics and birth control methods among patients at four SBHCs in New Mexico. Data was collected in November 2016 and supported the purpose of the TEMPO study, which was to evaluate a brief intervention for unintended teenage pregnancy. Teens recruited for the TEMPO study were required to be between the ages of 13 to 19, report unprotected vaginal sex in the past year, and not currently have a Long-Acting Reversible Contraceptive (LARC). Screening questions included these numerous public health-related topics, such as the number of servings of vegetables and fruits eaten per week and questions related to sexual health. The queries related to sexual experiences included asking teens whether or not they have had vaginal intercourse and if so, whether it was protected or unprotected sex. Teens who reported unprotected sex were deemed eligible for the study. Those interested in participating were approved for the study and asked to provide follow-up contact information. Parents provided permission to participate in the TEMPO study. The youth provided assent. After permission and assent was obtained from the teen, a baseline survey was administered via iPad, that specifically focused on questions related to sexual experiences and sexual health topics. Baseline questions included demographic questions, gender identity, sexual orientation, reasons for visits, reproductive practices, and birth control methods. Responses included fixed and scale options. Scales were based on 0–10, where 0 represented the lowest score for example, “Not confident at all,” and 10 represented the highest score, “Extremely confident”. Questions regarding reproductive practices were based on a 0–10 scale where 0 represents “Not important at all,” and 10 represents the highest score, “Extremely important”. After completion of the baseline survey, participants were randomized into one of two groups: intervention or control for the larger study. This paper presents only baseline data from patients at SBHCs enrolled in the TEMPO study. Descriptive statistics were calculated, including means and standard deviations for continuous variables and categorical frequencies. The mean scores presented are based on a scale of 0–10.

## 3 Results

Table 1 presents the percentage of responses from patients involved in the TEMPO study and baseline data collection.

**Table 1:** Characteristics of New Mexico patients at SBHCs (*n* = 264).

Category	Variable	Percentage (%)	Frequency (*n*)
Race and ethnicity	Hispanic or Latino	74.60%	197
	Other	42.80%	113
	White	39.80%	105
	American Indian Alaska Native	12.50%	33
	Black or American African–American	8.30%	22
	Native Hawaiian or other Pacific Islander	1.50%	4
	Asian	2.30%	6
Age	14	3.80%	10
	15	19.70%	52
	16	19.30%	51
	17	24.20%	64
	18	20.10%	53
	19	12.90%	34
Sex at birth	Male	28.80%	76
	Female	71.20%	188
	Intersex	0%	0
	Other	0%	0
Gender identity	Male	28.40%	75
	Female	70.50%	186
	Other	0.80%	2
	Two-spirit	0.40%	1
	Transgender	0%	0
	Genderqueer/gender non-conforming	0%	0
Sexual orientation	Heterosexual	88.20%	232
	Bisexual	8.40%	22
	Pansexual	1.90%	5
	Not sure	1.10%	3
	Gay	0.80%	2
	Lesbian	0.40%	1
Reasons for visit	Birth control	39.80%	105
	Regular physical	17.00%	45
	Other	17.00%	45
	STD check	15.50%	41
	Pregnancy check	11.40%	30
	Sick	7.20%	19
	Sports physical	5.70%	15
	Emergency birth control	4.20%	11
Vaginal sex	Ever had vaginal sex	100%	264
	Ever pregnant, gotten someone pregnant	5.30%	14
	Past three months vaginal sex	87.10%	230
	Past three months, times vaginal sex condom	84.30%	194

A total of 264 teens were included in the baseline data collection at SBHCs in New Mexico; five were omitted due to missing data. Most teens were heterosexual females between the ages of 17 and 18. Reasons for visiting the SBHC varied, from regular physicals (39.8%) to emergency birth control (4.2%). Regarding sexual activity, 87.1% reported vaginal sex in the past three months, and 84.3% reported they used a condom during vaginal sex in the past three months.

Table 2 presents reproductive practices, importance of reproductive behaviors, and methods of birth control.

**Table 2:** Reproductive practices of SBHC patients^a^.

Category	Variable	Mean or %	Standard deviation (SD)
Reproductive practices (*n* = 194)	Past three months, vaginal sex, no condom	15.7%	–
	Past three months, vaginal sex no pill, shot, patch ring, implant methods of birth control	39.2%	–
	In the past three months, the number of times sex, no birth control, pill, shot, patch, ring, implant	5.36	12.64
Reproductive importance (*n* = 264)	Importance to stop unprotected sex	7.67	2.84
	Confidence in stopping unprotected sex	7.4.3	2.74
	Readiness to stop unprotected sex	7.15	3.05
		Percentage	Frequency (*n*)
Methods of birth control (*n* = 264)	Condom	42.40%	112
	None	37.10%	98
	Birth control pills	15.50%	41
	Shot	6.80%	18
	Implants	5.30%	14
	Patch	0.40%	1
	Ring	0.40%	1
	IUD	1.50%	4

^a^ Note differences in sample size based on the number of respondents completing the questions. Due to variation of sample, “*n*” represents the actual number of respondents for each category in Table 2. These are based on 10point readiness scale.

Patients report having vaginal sex in the past three months an average of 7.62 times without a condom and 7.36 times with other birth control methods. In contrast, patients reported having vaginal sex 5.36 times with no birth control method. Patients reported a moderate to high level of importance to stop unprotected sex, confidence to stop unprotected sex, and readiness to stop unprotected sex (all mean scores >7.15 based on a 10-point scale). The most frequent method of birth control was condoms (42.4%) and the least was the patch or ring (0.4%).

## 4 Discussion

We explored student reproductive health practices at SBHCs in New Mexico. We found that the majority of eligible patients were females, and their sexual orientation varied from heterosexual, bisexual, pansexual, not sure, gay, and lesbian. While sexual minority groups must be included as a priority population for SBHCs, they represented a relatively small percentage of patients in the baseline data. The majority were Hispanic or Latino. This is consistent with what we would expect since the majority of patients at SBHCs were Hispanic.

Regarding birth control methods, just 62.9% of patients in this study report they used birth control the last time they had sex. In contrast, 86% of teens in the US report they used birth control the last time they had sex and less than 5% of teens in the US on birth control use the most effective types (12). The most common method of birth control among patients in this study was a condom, this is consistent with previous literature on US teens where condoms were reported by 97% of teen females followed by withdrawal and the pill (13).

Patients in this study are ready to improve their reproductive health practices and report high levels of readiness and confidence about using birth control methods in the future. While similar studies are not available for comparison, we feel that this signifies these patients are ready for behavior change and services provided through SBHCs. Previous studies indicate that increased access to reproductive health services addresses barriers and stigma associated with seeking reproductive health resources (6, 14, 15). Researchers have found that school-based sexual and reproductive health education is effective in promoting healthy reproductive practices in youth, including delaying first sexual intercourse, reducing the number of sexual partners, decreasing the frequency of unprotected sex, increasing condom and contractive use, and gains in academic achievement and performance (1, 3).

One indicator of reproductive health service utilization is teen pregnancy rates. While New Mexico still ranks 10th in the US for teen pregnancy, this is an improvement from previous years. Strides may be attributed to increases in education and accessibility to methods of contraception in a SBHC setting (16). New Mexico has also increased low-cost family planning services through public health and primary care sites. However, continued advocacy and support of SBHCs are needed to close the gap and reduce teen pregnancy rates in New Mexico.

Adolescent populations in New Mexico benefit from having SBHCs in their communities. By having access to an SBHC, adolescents not only have access to a wide variety of treatments and resources, but they also have the autonomy to pursue health care anonymously and independently of parents and guardians. NM SBHCs are often able to reach and assist teens with sexual health concerns when others cannot. They do so by providing easy access to free contraception, using teen-friendly communication styles, and being easily accessible during the school day, thereby eliminating real/perceived barriers to reproductive/sexual health care.

### 4.1 Limitations

Baseline data do not represent all youth seeking reproductive health services at SBHCs in New Mexico. These data provide a snapshot of the characteristics and choices. Moreover, findings from the larger TEMPO study were not analyzed or published due to staffing and leadership changes. Data were collected in November 2016 and since this time, there have been changes in the development of SBHCs, laws, and public health conditions related to the COVID-19 pandemic. Therefore, it is unclear how baseline responses changed as a result of the behavioral intervention. Racial and ethnic classifications are limited because the “other” category did not include specific information about what others represented. Responses are based on self-report data and subject to social desirability bias. For example, participants appear confident and ready to change behaviors, yet 37% use no birth control. Even with these limitations, this study provides the first-ever documentation of SBHC patients' reproductive characteristics in New Mexico.

### 4.2 Conclusions

Findings underscore the need for continued funding, policy, and advocacy around reproductive health and SBHCs. These efforts may be more beneficial in states like New Mexico where teen pregnancy rates are among the highest in the nation, and poverty and lack of medical insurance present gaps in access to services. New Mexico state laws that allow SBHCs to provide reproductive health services without parental permission (8) must be protected and continued.

Our findings present reproductive health behaviors among New Mexican patients attending schools with SBHCs. These behaviors are essential for policymakers and educators to consider as they navigate the complex landscape of consent, parental permission, reproductive rights, and health as a human right (4). Despite the United Nations Declaration that health is a human right (17), not every student or person in New Mexico has full access to services and resources that support and uphold this right. Moreover, continued efforts are necessary in New Mexico and throughout the world to meet the World Health Organization's Sustainable Development Goal Target 3.7 address sexual and reproductive health. This goal aims to ensure universal access to sexual and reproductive health services, and the integration of reproductive health into national strategies (18). SSBHCs in New Mexico and beyond must continue to reach students through innovative, on-site services that fill gaps in the current healthcare system. SBHCs are part of a global solution to lowering teen pregnancy and infant mortality rates and increasing access to care for all.

## Data Availability

The original contributions presented in the study are included in the article/Supplementary Material, further inquiries can be directed to the corresponding author.
